# ESR Essentials: uterine cancers—practice recommendations by the European Society of Urogenital Radiology

**DOI:** 10.1007/s00330-026-12705-2

**Published:** 2026-07-01

**Authors:** Roberta Valerieva Ninkova, Silvia Bottazzi, Yulia Lakhman, Benedetta Gui, Stefania Rizzo, Isabelle Thomassin Naggara, Lucia Manganaro, Stephanie Nougaret

**Affiliations:** 1 Department of Radiological, Oncological and Pathological Sciences, Sapienza–University of Rome, Rome, Italy; 2 Department of Experimental Medicine, Sapienza–University of Rome, Rome, Italy; 3 Dipartimento Diagnostica per Immagini e Radioterapia Oncologica, Fondazione Policlinico Universitario A. Gemelli IRCCS, Rome, Italy; 4 Department of Radiology, Memorial Sloan Kettering Cancer Center, New York, NY USA; 5 Imaging Institute of Southern Switzerland, Ente Ospedaliero Cantonale (EOC), Lugano, Switzerland; 6 Faculty of Biomedical Sciences, Università della Svizzera Italiana (USI), Lugano, Switzerland; 7 Imageries Radiologiques et Interventionnelles Spécialisées (IRIS), APHP Sorbonne Université Hopital Tenon, Paris, France; 8 Sorbonne Université, GRC Endometriose, Paris, France; 9 INSERM, PINKCC Lab, Montpellier Cancer Research Institute, University of Montpellier, Montpellier, France; 10 Department of Radiology, University of Montpellier, Montpellier, France

**Keywords:** Cervical neoplasms, Endometrial neoplasms, Leiomyosarcoma, Magnetic resonance imaging, Uterine neoplasms

## Abstract

**Abstract:**

Uterine cancers include endometrial cancer (EC), cervical cancer (CC), and uterine sarcomas, with leiomyosarcoma being the most common subtype. Each tumour type has distinct biological behaviour, imaging features, and treatment approaches. Radiologists must be familiar with normal uterine anatomy and its variations, as well as key imaging characteristics and common patterns of tumour spread to ensure accurate staging and optimal treatment selection. MRI is the imaging modality of choice for evaluating uterine malignancies due to its excellent soft tissue contrast and multiplanar capabilities. Accurate assessment requires dedicated multiparametric protocols, as recommended by the European Society of Urogenital Radiology (ESUR), including high-resolution T2-weighted imaging, diffusion-weighted imaging (DWI), and dynamic contrast-enhanced (DCE) sequences when appropriate. 18-Fluorodeoxyglucose Positron Emission Tomography (FDG-PET) complements MRI in patients with CC by providing the highest accuracy for detecting lymph-node and distant metastases. CT is mainly used for systemic evaluation. This article presents a practical guide for radiologists, describing the main clinical indications, patient preparation, and tailored MRI protocols. It also highlights key imaging findings that should be evaluated and reported.

**Key Points:**

- MRI is the preferred imaging modality for evaluating uterine malignancies due to its superior soft tissue contrast and multiplanar capabilities.
- Common clinical indications include MRI staging of endometrial and cervical cancers, assessment of eligibility for fertility preservation, and characterisation of indeterminate myometrial lesions.
- Adherence to established imaging guidelines is strongly recommended to ensure high-quality images as well as complete and clinically relevant radiology reports.

## Key recommendations

- In cervical cancer, MRI should be used to assess local tumour extent and guide treatment selection; it is mandatory to confirm eligibility for fertility-sparing surgery (level of evidence: moderate) [1].
- MRI should be used to reliably determine the maximal tumour size, measure tumour–internal os distance, and to evaluate parametrial invasion. These imaging features must be assessed and evaluated for optimal treatment selection, including fertility preservation (level of evidence: high) [2].
- In patients with endometrial cancer, MRI should be utilised for evaluating myometrial invasion and cervical stromal involvement (level of evidence: high).
- MRI is mandatory in patients with endometrial cancer prior to fertility-sparing treatment to confirm that the disease is confined to the endometrium (level of evidence: moderate).
- MRI should be performed to determine whether a tumour originates from the endometrium or cervix when pathology is inconclusive (level of evidence: moderate) [3–5].
- Consider MRI the imaging modality of choice for evaluating indeterminate myometrial lesions, such as differentiating uterine leiomyomas from leiomyosarcomas (level of evidence: high) [6].

## Introduction

Cervical cancer (CC) and endometrial cancer (EC) are the most common uterine malignancies, while leiomyosarcoma is much rarer. These tumours differ significantly in their biology, imaging features, and treatment implications. Proper staging of CC and EC and accurate diagnosis of leiomyosarcoma are essential for optimal treatment planning.

**Fig. 1:**
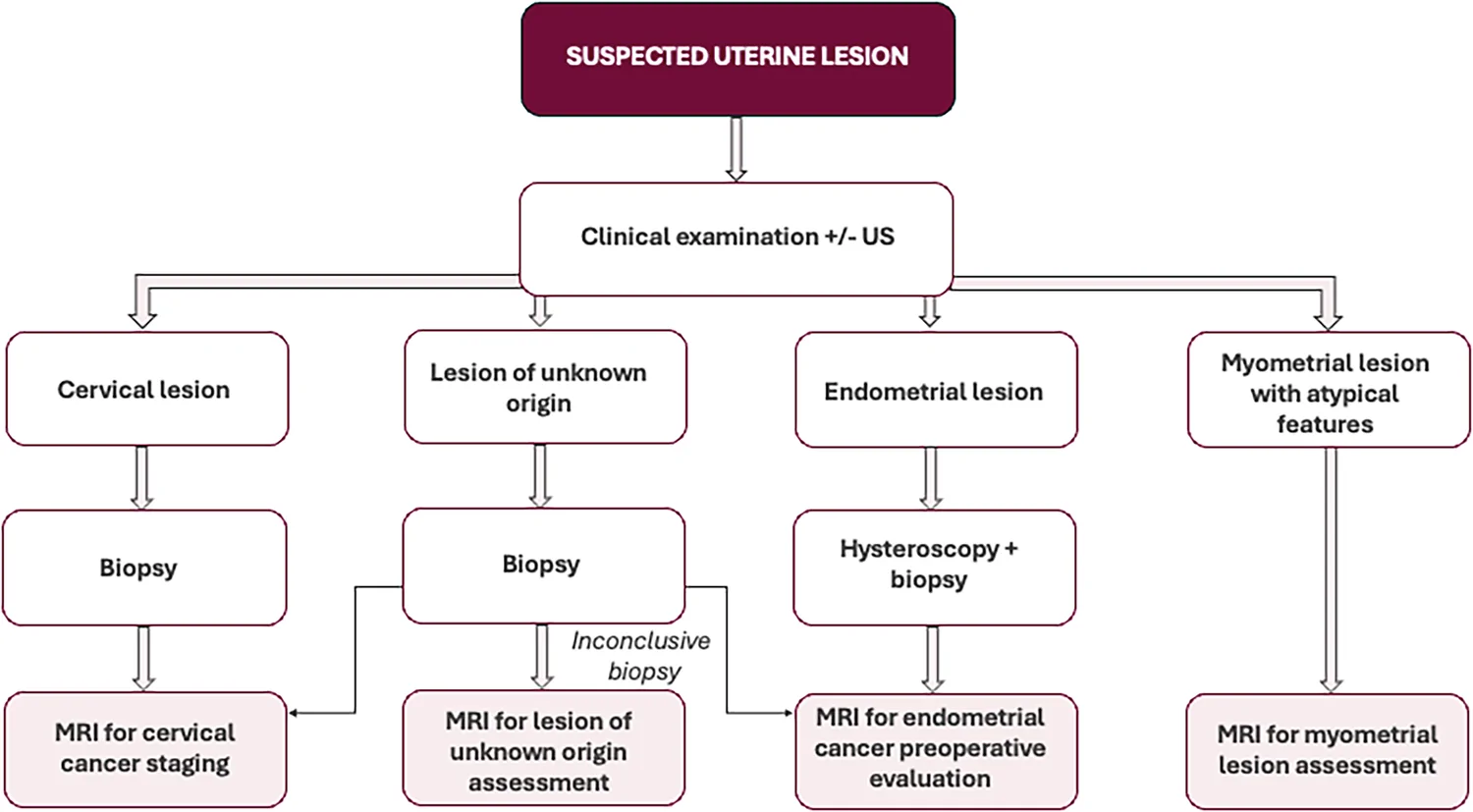
Workflow illustrating the most common clinical scenario for uterine cancers. US, ultrasound. Diagnostic algorithm for the evaluation of a suspected uterine lesion. Following clinical examination and/or ultrasound, lesions are classified as cervical, endometrial, myometrial with atypical features, or of uncertain origin. Cervical lesions undergo biopsy followed by MRI for staging. Endometrial lesions undergo hysteroscopy with biopsy followed by MRI for preoperative endometrial cancer assessment, particularly when evaluating eligibility for fertility-sparing treatment. In cases of myometrial lesions with atypical ultrasound features, MRI is performed for further lesion characterisation. MRI also serves as a problem-solving modality in the uncommon setting of an inconclusive biopsy or persistent uncertainty regarding lesion origin

MRI is the modality of choice for initial staging of CCs and ECs, including eligibility for fertility-sparing treatments. It is also used for the characterisation of indeterminate myometrial masses and assessment of known or suspected locoregional recurrence [7, 8]. A schematic workflow with the most common clinical scenario is shown in Fig. 1.

The European Society of Urogenital Radiology (ESUR) has published dedicated guidelines for MRI acquisition and interpretation in uterine cancers. Adherence to these guidelines is strongly recommended, as they include specific sequences to assess clinically relevant features, such as parametrial invasion (PMI) in CC, which shifts treatment from surgery to chemoradiation, or criteria for fertility-sparing management in early-stage CCs or ECs [8, 9].

The following sections summarise the most recent ESUR recommendations for MRI in uterine cancers.

## MRI protocols and structured report templates for uterine malignancies

The MRI protocols for uterine malignancies and examples of structured report templates are summarised in Tables 1 and 2, respectively.

**Table 1:**
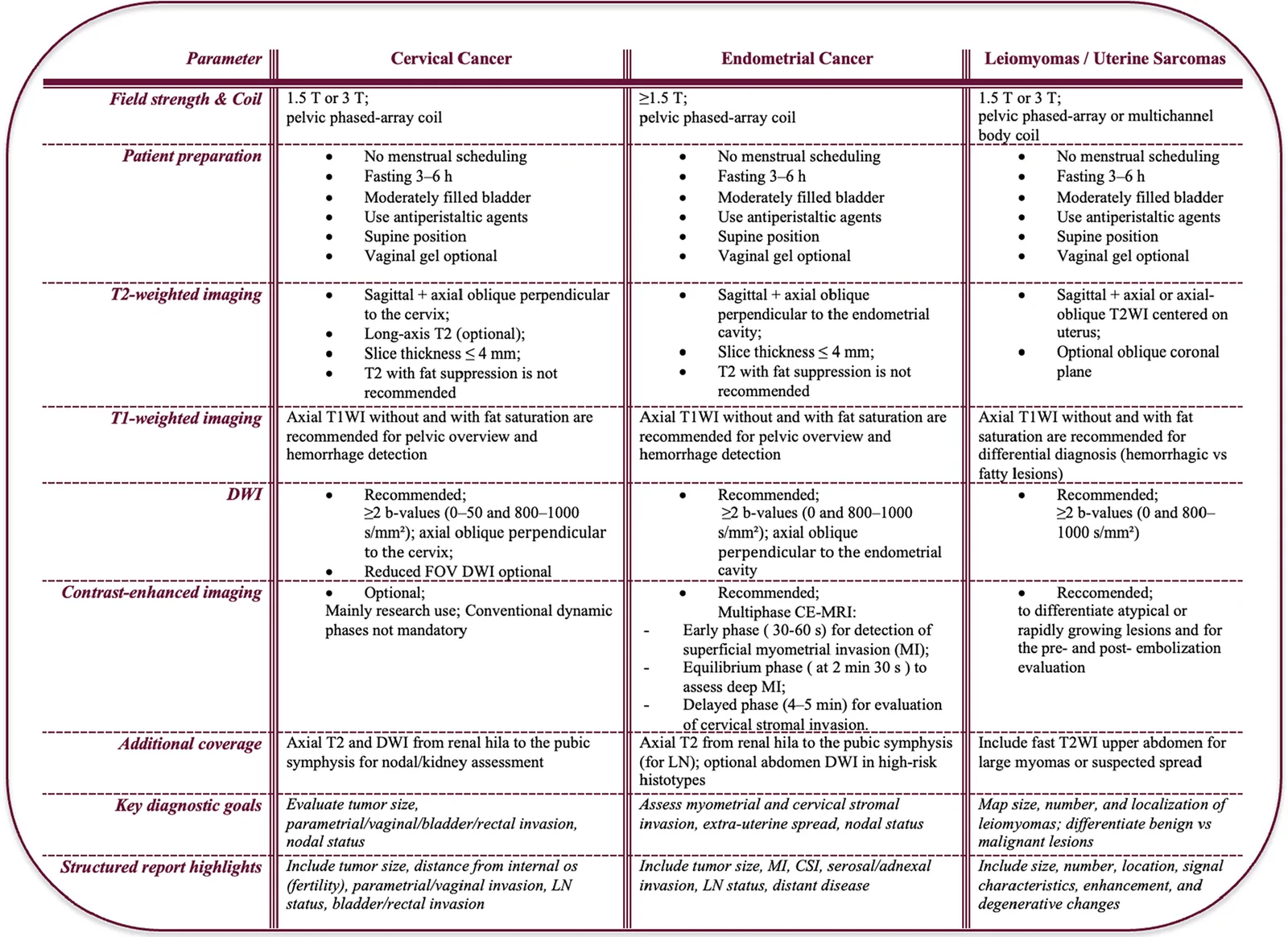
Comparison of MRI protocols for uterine malignancies

*FOV* field of view, *DWI* diffusion-weighted imaging, *LN* lymph node, *MI* myometrial invasion, *CSI* cervical stromal invasion, *CE-MRI* multiphase MRI

**Table 2:**
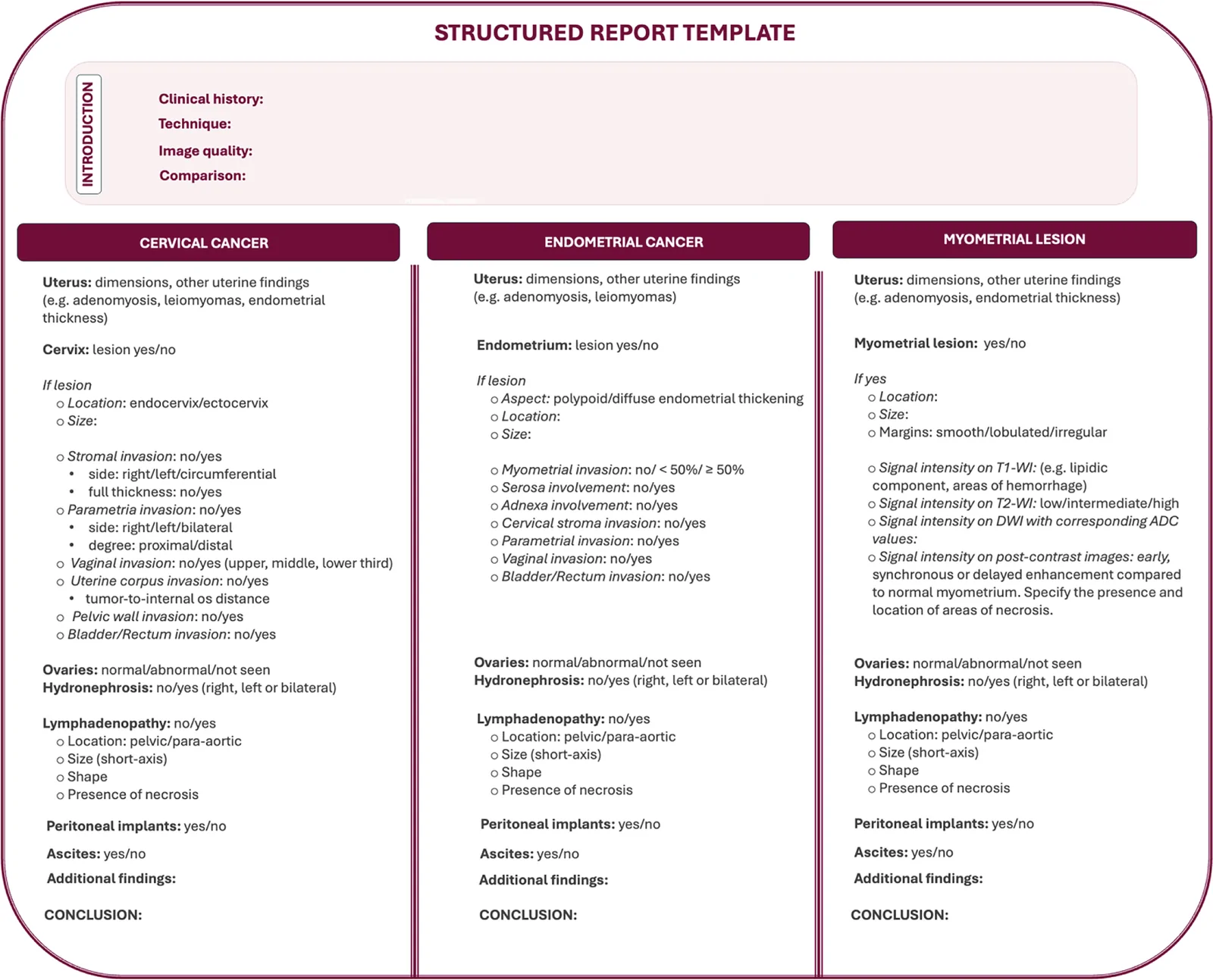
Examples of structured report templates

## Cervical cancer

CC is the fourth most common malignancy in women [10]. Persistent infection with human papillomavirus (HPV) is the main aetiologic factor, particularly in addition to HIV infection. The most common histological type is squamous cell carcinoma (70–80%), followed by adenocarcinoma and other less common subtypes such as neuroendocrine tumours [11].

Prognosis is strongly influenced by the anatomic disease extent at diagnosis, particularly by the location and number of metastatic LNs [12].

Imaging plays a central role in treatment planning by guiding patient selection for radical surgery, fertility-sparing procedures, or chemoradiotherapy [2].

MRI is valuable for local staging and tumour response and recurrence evaluation [2, 13]. FDG-PET, fused with CT or MRI, is recommended to evaluate LN and distant metastases. FDG-PET is also useful for assessing metabolic response to chemoradiotherapy and for detecting recurrent disease [14].

The following sections summarise the key MRI features that are fundamental for staging and treatment selection.

### Tumour imaging features and dimensions

**Fig. 2:**
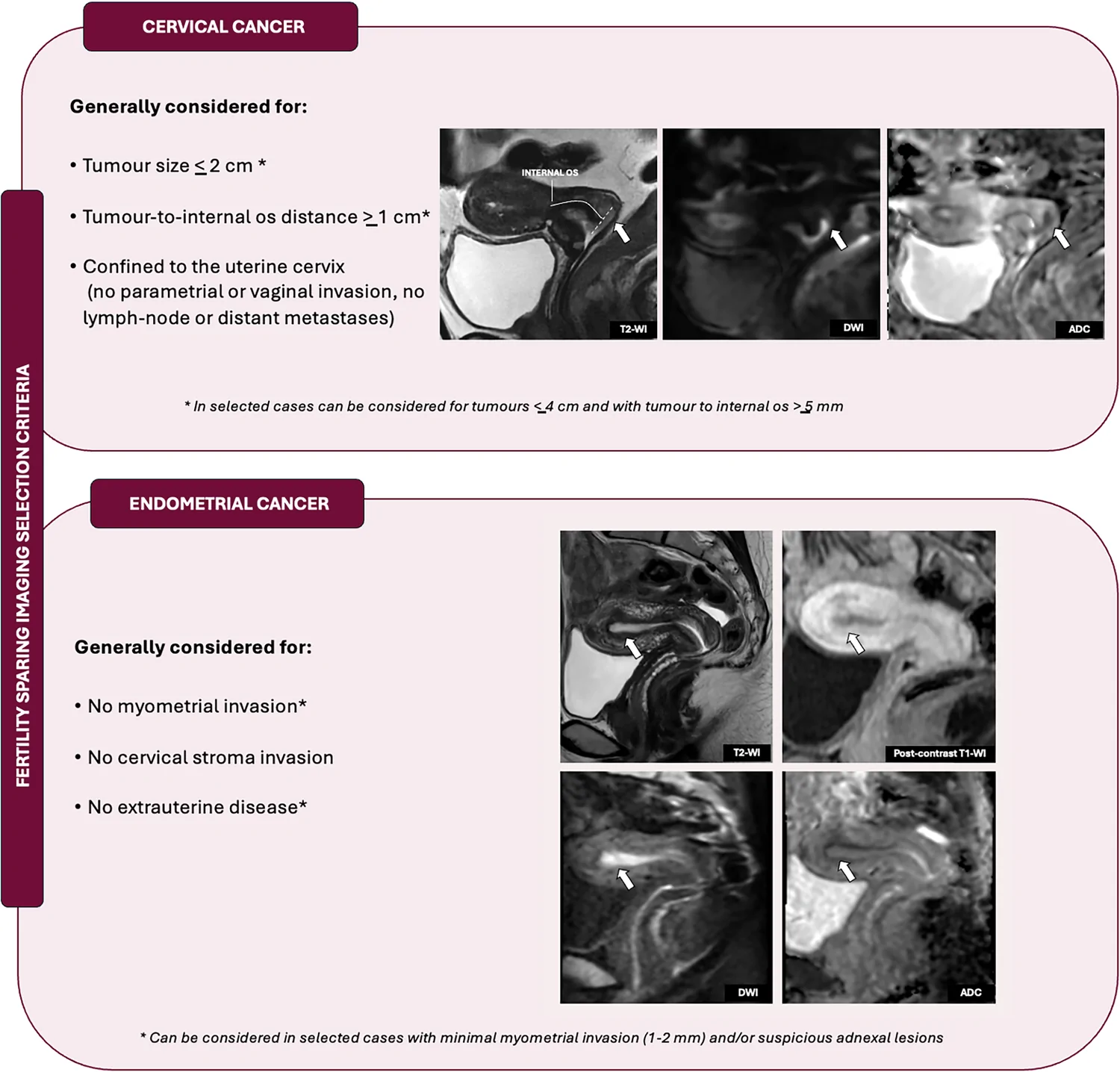
Imaging selection criteria for fertility-sparing treatment in cervical and endometrial cancer

In the 2018 Federation of Gynecology and Obstetrics (FIGO) system, stage IB is now divided into three groups by the greatest tumour dimension: IB1 (≤ 2 cm), IB2 (> 2–4 cm), and IB3 (> 4 cm). This change reflects the prognostic value of tumour size and guides treatment [12]. On MRI, CC typically demonstrates intermediate signal intensity (SI) on T2-weighted images (T2-WI), high signal on high-b-value DWI and low ADC values. Less common histological subtypes may show atypical features. For example, gastric-type adenocarcinoma and mucinous adenocarcinoma often demonstrate less prominent or absent diffusion restriction [11]. The combination of T2-WI and DWI improves the delineation of tumour margins and allows accurate measurement of the greatest tumour dimension [11, 15]. Sagittal T2-WI are particularly useful for assessing tumour length and the tumour-to-internal os distance, key for determining fertility-sparing eligibility. Fertility-sparing surgery is generally considered for squamous cell carcinoma or adenocarcinoma confined to the cervix with tumour size ≤ 2 cm and tumour-to-internal os distance ≥ 1 cm (Fig. 2) [9].

### Parametrial invasion

**Fig. 3:**
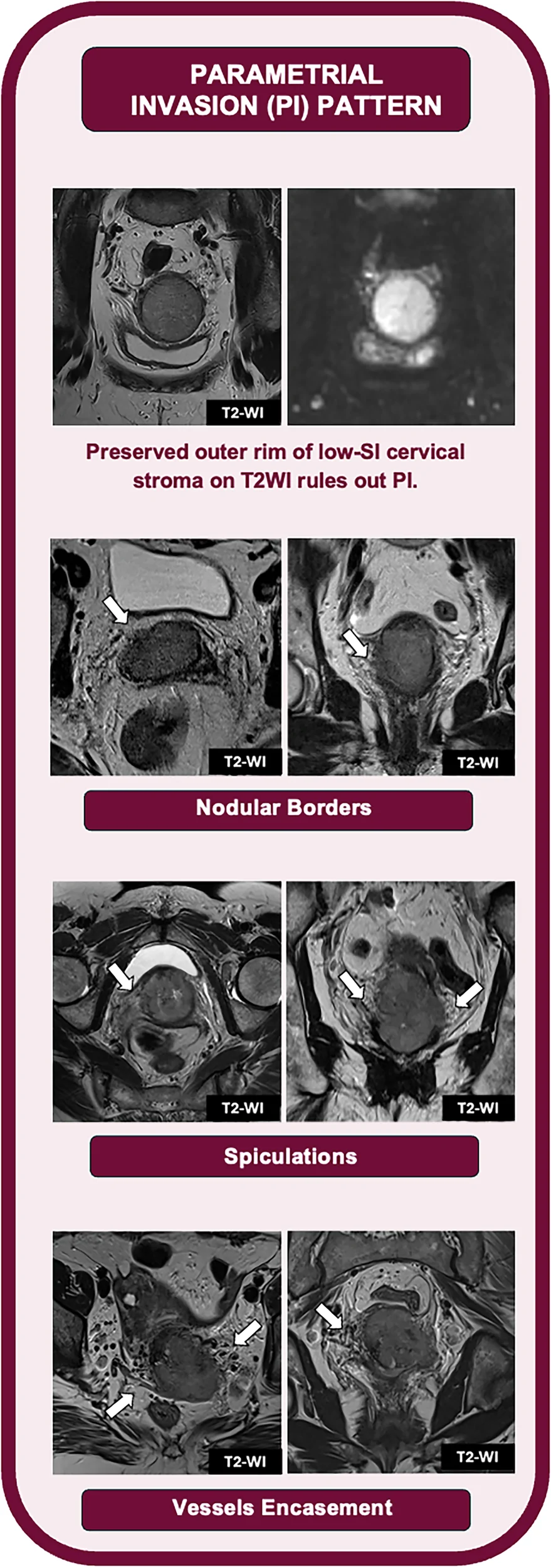
Parametrial invasion (PI) patterns on MRI

Assessment of PMI is crucial, as its presence upgrades the disease to stage IIB and shifts management from surgery to chemoradiation. High-resolution short-axis T2-WI, acquired perpendicular to the cervix, is superior to a conventional axial plane for evaluating the tumour-parametria interface [8]. Preservation of the low-SI fibrous cervical stromal ring on T2-WI, which corresponds to dense fibromuscular stromal tissue on histology, excludes PMI with a high negative predictive value. Full-thickness cervical stromal invasion (CSI) alone does not indicate parametrial spread. PMI is diagnosed when full-thickness invasion coexists with spiculated or nodular tumour projections into the parametrium, without or with encasement of parametrial vessels (Fig. 3) [16]. The addition of DWI increases accuracy by improving the delineation of tumour margins, helping to differentiate tumour from fibrosis or oedema [8].

### Vaginal invasion, hydronephrosis and pelvic wall invasion

Stage IIA refers to tumour spread into the upper two-thirds of the vagina without PMI. Tumours ≤ 4 cm (IIA1) are managed surgically, whereas tumours > 4 cm (IIA2) are treated with chemoradiotherapy. Notably, upper vaginal involvement precludes fertility-sparing surgery. Stage IIIA indicates invasion of the lower third of the vagina. On T2-WI, vaginal infiltration appears as a disruption of the hypointense vaginal wall by intermediate-signal tumour. On sagittal T2-WI, a horizontal line drawn approximately at the level of the bladder neck can be used as a practical imaging landmark to distinguish the upper two-thirds and the lower one-third of the vagina.

Ureteral and pelvic sidewall invasion (stage IIIB) refers to the distal ureter invasion, causing hydronephrosis, and/or direct involvement of the pelvic sidewall. Loss of the fat plane between tumour and pelvic sidewall muscles (including the obturator internus and piriformis) and/or pelvic vessels (including the internal iliac vessels and their major branches) is suggestive of pelvic sidewall invasion [14]. When present, it may preclude salvage surgery, such as pelvic exenteration, for residual or recurrent disease because complete tumour resection with negative margins may not be feasible [17].

### Lymph-node status

A key update to the 2018 FIGO revision was the inclusion of pelvic and para-aortic LN involvement as stage IIIC, reflecting their important prognostic role. MRI evaluates LN metastases using morphologic criteria, including a short-axis diameter ≥ 10 mm, rounded shape, irregular borders, and heterogeneous SI. This approach provides moderate sensitivity. but high specificity. Lowering the size threshold to 8 mm may improve sensitivity but increases false positive findings [18]. FDG-PET currently provides the highest diagnostic performance for nodal staging. It is recommended for patients with tumours > 4 cm or higher stage disease, where the risk of LN metastases is greater [8].

### Pelvic and extrapelvic spread

Stage IVA indicates direct invasion into the mucosa or lumen of adjacent pelvic organs, such as the urinary bladder or rectum, whereas stage IVB denotes distant metastases. On MRI, bladder or rectal invasion is diagnosed when the intermediate-signal tumour breaches the low-signal muscular walls and extends into the mucosa or lumen. The loss of the fat plane between the tumour and these organs should not be overinterpreted as invasion. Bullous oedema of the bladder must also be distinguished from true mucosal involvement by tumour, as it may also be caused by infection or inflammation.

FDG-PET/CT or PET/MRI is recommended for detecting distant spread. Accurate detection of distant metastases is essential for identifying stage IVB disease, which is primarily managed with systemic chemotherapy, without or with palliative radiotherapy [8].

The MRI features described are schematically summarised in Table 3.

**Table 3:**
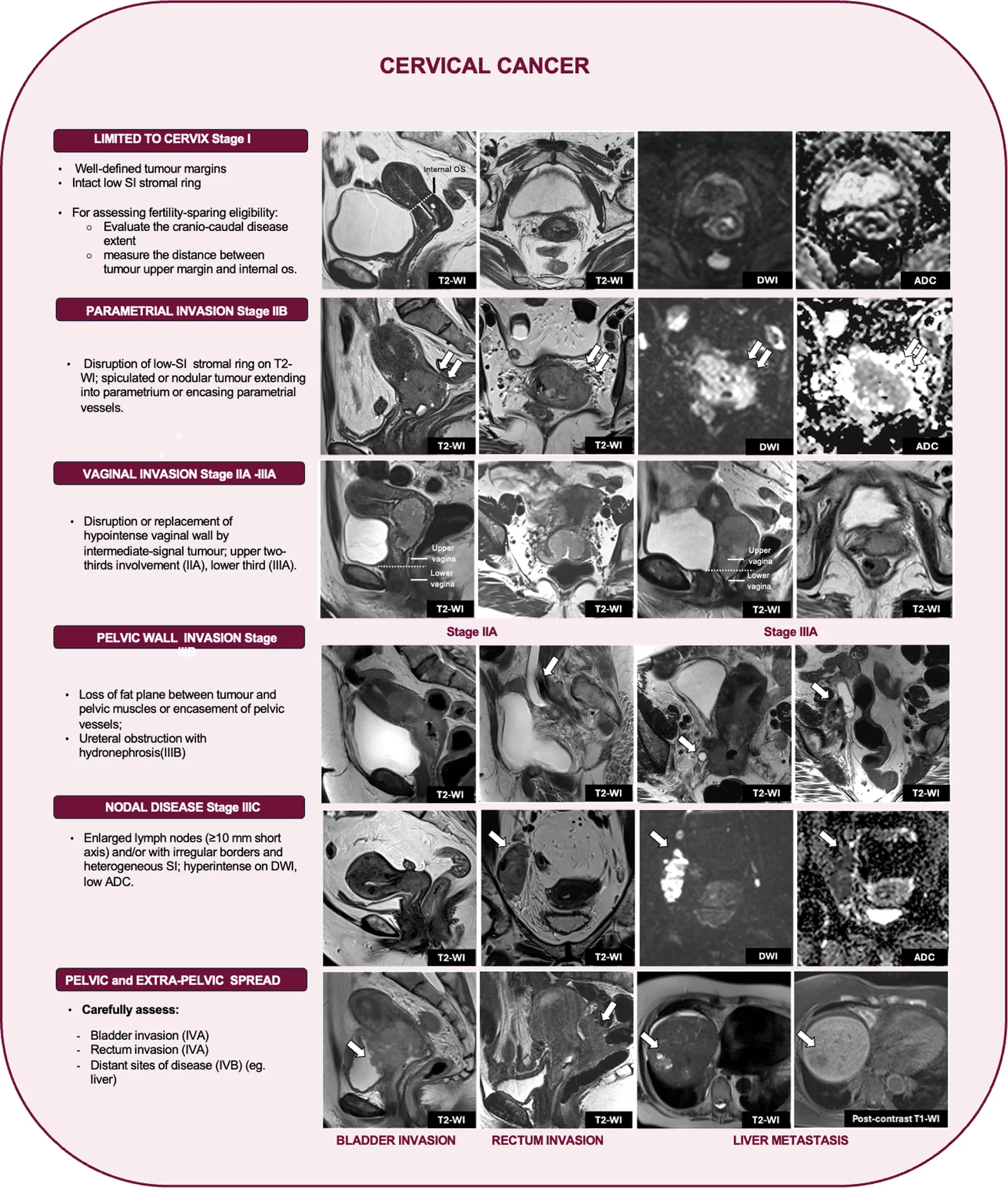
Key imaging findings in cervical cancer

*SI* signal intensity, *DWI* diffusion-weighted imaging, *ADC* apparent diffusion coefficient

## Endometrial cancer

EC is the sixth most common malignancy in women [10]. Increased exposure to oestrogens is the primary risk factor. Lynch syndrome is responsible for a minority of cases [11]. Although most ECs occur after menopause, up to 7% are diagnosed in women younger than 45 years, particularly those with polycystic ovary syndrome, which is associated with elevated endogenous oestrogen levels. Surgery is the primary treatment for EC [9, 11].

The FIGO staging system for EC was recently revised, introducing new substages and incorporating parameters beyond the traditional anatomic tumour extent. These include tumour histology and grade, LN micrometastases, lymphovascular space invasion and molecular status [19]. The incorporation of histologic features and molecular classification reflects their prognostic and therapeutic implications. For example, POLE-mutant and mismatch-repair-deficient tumours are associated with better prognosis and potential eligibility for adjuvant immunotherapy [20].

Despite these additions, MRI remains central in treatment planning—including fertility-sparing selection and evaluation of suspected recurrence (Fig. 2). MRI can also be used for EC diagnosis, if biopsy is not feasible, and to determine tumour origin (endometrial vs endocervical) in the rare case of inconclusive pathology [7, 21].

Below are described the key MRI parameters that should be systematically assessed and reported.

### Tumour imaging features and dimensions

On MRI, EC may appear as a mass-forming lesion, a polypoid lesion, or diffuse endometrial thickening. Because endometrial thickness varies with menopausal status and phase of the menstrual cycle, this information should always be available at the time of interpretation.

**Fig. 4:**
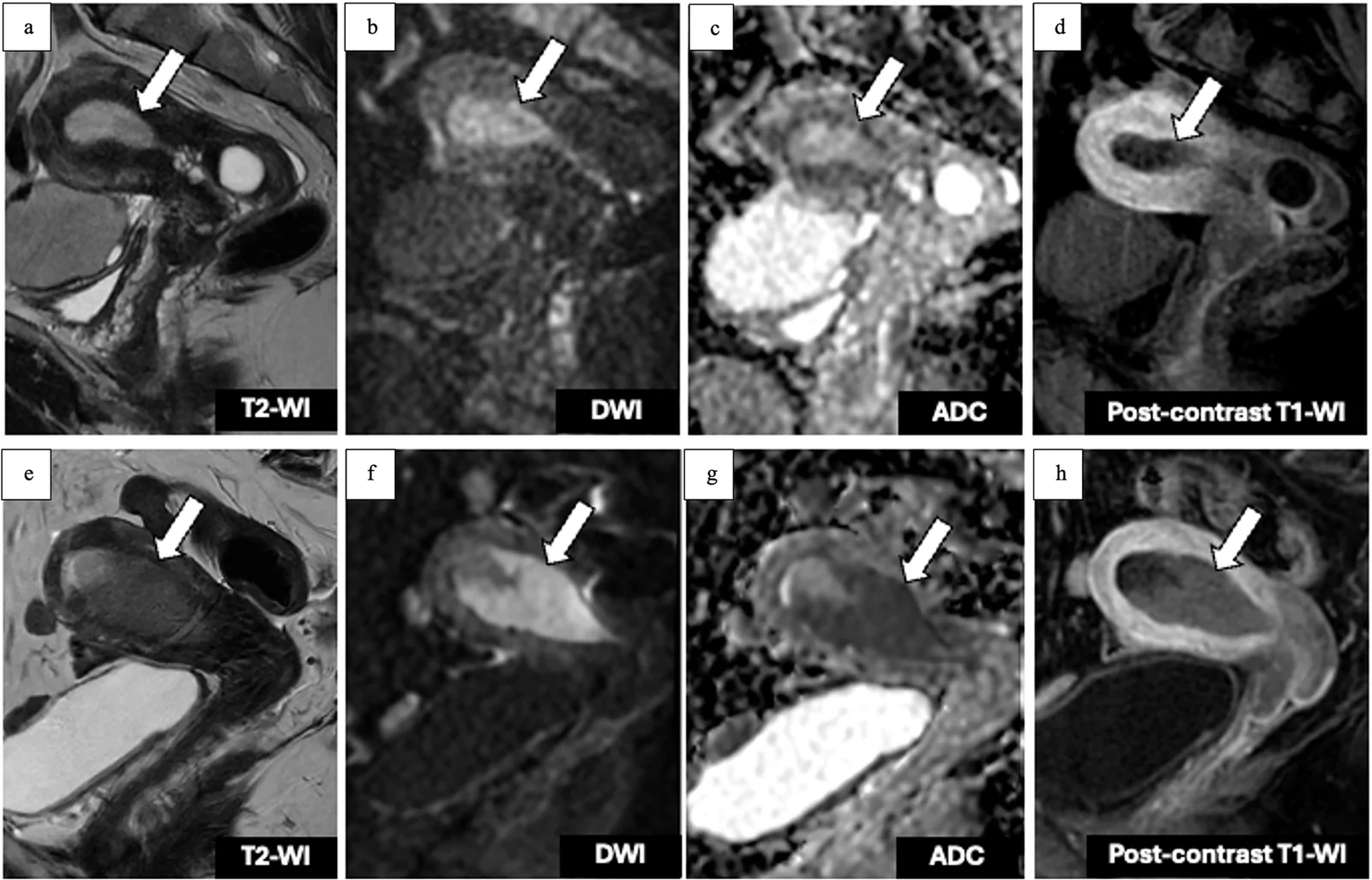
Typical imaging features of endometrial hyperplasia and endometrial carcinoma. Upper row: sagittal T2-WI (**a**), DWI (**b**), ADC map (**c**) and post-contrast images (**d**) demonstrate diffuse endometrial thickening with high signal intensity on T2-WI and DWI, and corresponding intermediate-to-high ADC values (> 1.000 × 10⁻³ mm²/s), consistent with endometrial hyperplasia. Lower row: sagittal T2-WI (**e**), DWI (**f**), ADC map (**g**) and post-contrast images (**h**) illustrate the characteristic features of endometrial carcinoma, including intermediate signal intensity on T2-WI, marked diffusion restriction (low ADC values), and relative hypovascularity on post-contrast images

Compared with the homogeneously high T2-SI of normal endometrium, EC typically demonstrates intermediate, often heterogeneous SI and diffusion restriction. While the normal endometrium enhances less than the myometrium in the early phases, and similar in the later phases, EC usually hypoenhances compared with the myometrium on all contrast-enhanced phases [7]. Less common histological subtypes may demonstrate atypical enhancement patterns [15]. Some tumours are isointense to the surrounding myometrium on T2-WI; in such cases, DWI and post-contrast images are particularly helpful for tumour delineation. EC usually shows lower ADC values (< 1.0 × 10⁻³ mm²/s) than endometrial hyperplasia or benign polyps, aiding in differential diagnosis, as shown in Fig. 4 [22]. Histopathological evaluation remains the gold standard for EC diagnosis.

MRI is also valuable in rare situations when pathologic evaluation cannot conclusively distinguish tumour origin. An endometrial origin should be suspected when the lesion is centred in the endometrial cavity above the internal os and has an elongated configuration. In contrast, CC is typically centred below the internal os, has a round or oval morphology, and invades into or through the cervical stroma into the parametria [23].

### Myometrial invasion (MI)

The presence and depth of MI on MRI are important imaging features, as tumour substages in the 2023 FIGO staging system are assigned based on whether MI is absent, superficial (< 50%) or deep (≥ 50%) [20]. As FIGO 2023 requires MRI to exclude any degree of MI, the updated ESUR guidelines on EC recommend the use of multiphase contrast-enhanced (CE) for accurate assessment, as shown in Table 1. In fact, in low-risk histologies, absence of MI may allow omission of sentinel node mapping and sampling and is an eligibility criterion for fertility-sparing treatment for patients with low-grade endometrioid adenocarcinoma desiring fertility [22]. However, the delayed phase may be omitted if protocol optimisation is required. In patients with contraindications to gadolinium, staging should rely on high-resolution T2-WI and DWI, with the acknowledgement that confidence may be lower for excluding superficial myometrial invasion.

**Fig. 5:**
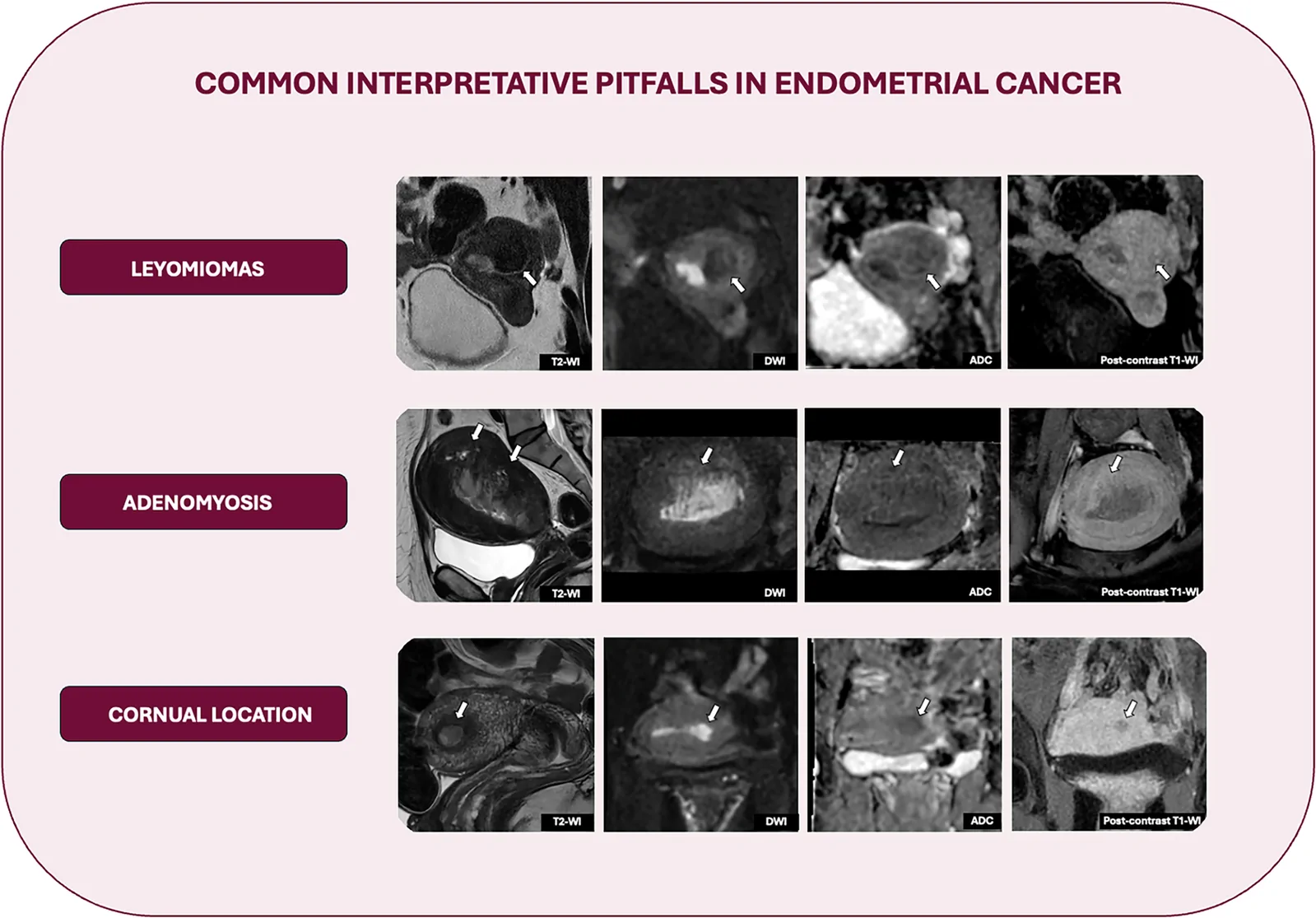
Common pitfalls in the imaging interpretation of endometrial cancer

On early-phase CE-MRI (< 60 s), an intact subendometrial enhancement (uninterrupted linear early enhancement of inner myometrium) excludes MI. However, this sign is visible in only 10–60% of cases. Other features, such as the smooth interface between the myometrium and tumour on T2-WI, DWI, and equilibrium-phase CE-MRI, are also useful for excluding MI [21, 22]. MI is suspected when the smooth endometrium-junctional zone (JZ) interface is interrupted by tumour SI extending into the myometrium. When MI is present, its depth can be optimally assessed combining T2-WI, DWI, and equilibrium-phase CE-MRI (120–180 s), acquired in axial-oblique and sagittal planes, in accordance with current guidelines (Table 1). Adenomyosis, leiomyomas and tumour in the cornual regions are common pitfalls that may complicate assessment and should always be reported (Fig. 5) [7].

### Cervical stromal invasion

CSI should be distinguished from tumour protrusion into the endocervical canal, as CSI is considered stage II disease. Normal cervical stroma has low SI on T2-WI, lacks diffusion restriction and demonstrates progressive homogeneous enhancement, best seen on delayed-phase images, in contrast to the relative hypoenhancement of the tumour. CSI is diagnosed when the tumour signal disrupts the smooth interface between the endocervical canal and cervical stroma. By contrast, tumour protrusion into the endocervical canal is characterised by expansion of the canal and thinning of the cervical stroma, with the preservation of the smooth interface [22].

### Extrauterine extension (serosa, adnexa, vagina, parametrium, bladder, rectum)

Most EC cases present at an early stage due to abnormal vaginal bleeding. Nevertheless, evaluating extrauterine spread is crucial, as advanced disease is operable only when complete resection is achievable [20]. Serosa involvement is suggested by disruption of the T2-hypointense outer uterine border and contour irregularity [24].

Adnexal metastases (Stage IIIA) are not uncommon and should be carefully evaluated; however, distinguishing them from a synchronous ovarian tumour can be challenging. A synchronous tumour should be suspected in early-stage EC with minimal or absent MI [24]. Detecting adnexal lesions is crucial before fertility preservation, as conservative treatments, such as hysteroscopy and progestins, do not address adnexal disease.

Vaginal and parametrial invasion (Stage IIIB) and invasion of the bladder and bowel mucosa (Stage IVa) are evaluated with approaches similar to those of CC.

### Lymph-node status

In the 2023 FIGO system, metastases to pelvic and/or para-aortic LNs are classified as Stage IIIC, whereas nodes above the renal vessels are considered distant metastases (Stage IVB). The system also distinguishes between nodal micrometastases (< 2 mm) and macrometastases in the pelvic and para-aortic regions. LN evaluation on MRI follows the same morphological criteria described for CC [25].

### Peritoneal spread

The presence of abdominal peritoneal metastases (beyond pelvis) must be clearly reported as they indicate stage IVB disease, whereas peritoneal metastases confined to the pelvis are considered stage IIIB2. DWI through the abdomen and pelvis is useful for detecting and mapping peritoneal dissemination [21]. Its extent and distribution influence treatment decisions and should be carefully documented in the report [20].

The MRI features described are schematically summarised in Table 4.

**Table 4:**
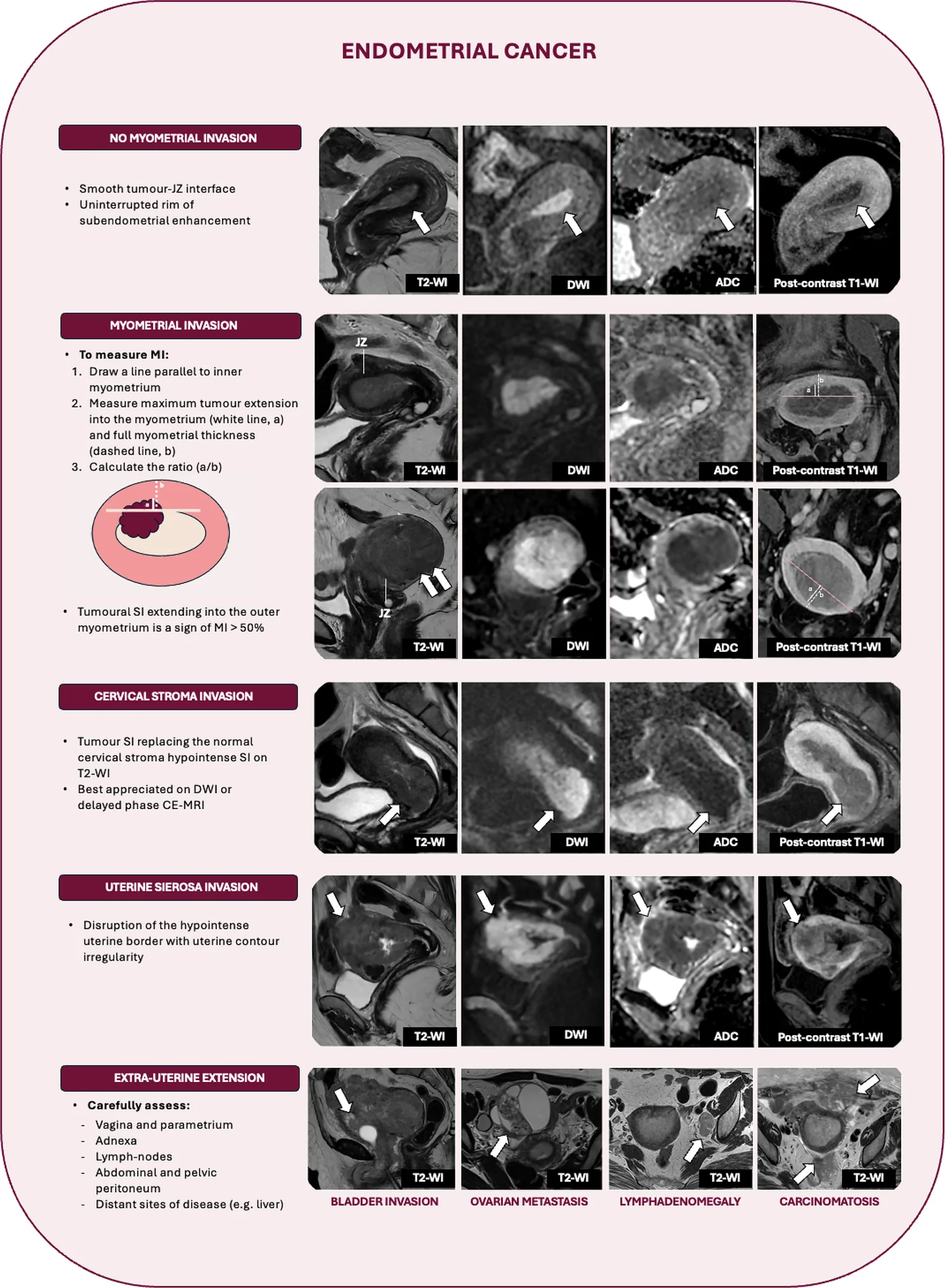
Key imaging findings in endometrial cancer

*JZ* junctional zone, *SI* signal intensity, *MI* myometrial invasion, *CE-MRI* multiphase MRI

## Leiomyomas and leiomyosarcomas

Leiomyomas are the most common gynaecological tumours, occurring in up to 80% of women over 50 years. By contrast, leiomyosarcomas are rarer, accounting for 3–7% of primary malignant uterine tumours [26–28]. Leiomyosarcomas are predominantly diagnosed in postmenopausal women [27], and only 2% arise from pre-existing leiomyomas [29].

Accurate differentiation between leiomyomas and leiomyosarcomas is critical, as symptomatic leiomyomas may be managed either surgically or conservatively, whereas leiomyosarcomas require prompt radical surgery because of their aggressive biology and poor prognosis. Preoperative identification of uterine masses suspicious for leiomyosarcoma is important because inadvertent morcellation of an unsuspected malignancy may cause tumour dissemination, while accurate risk stratification can still support minimally invasive surgery for women with benign leiomyomas [26]. Typical leiomyomas appear as round lesions with smooth or lobulated margins and homogeneously low SI on T2-WI and DWI, and avid enhancement. However, various types of degeneration (hyaline, cystic, myxoid, or red degeneration) and histologic variants (e.g., cellular leiomyoma, lipoleiomyoma) can result in atypical MRI appearances and make reliable distinction from leiomyosarcoma challenging [26, 29].

Research in this area is ongoing. However, to address this challenge, several MRI-based approaches have been proposed, incorporating different imaging features.

**Fig. 6:**
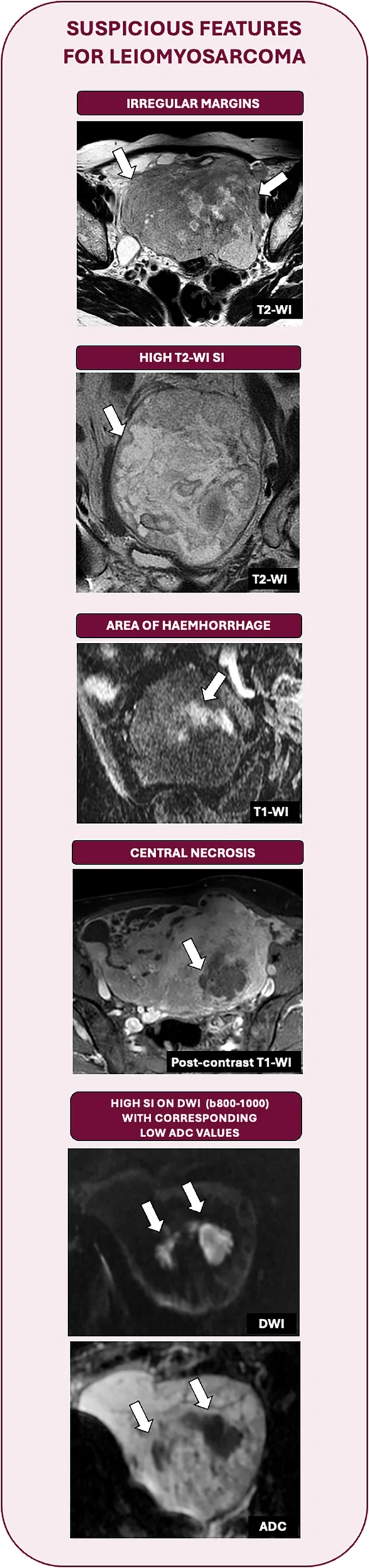
Suspicious imaging features for uterine leiomyosarcoma. SI, signal intensity

In addition to peritoneal and LN metastases, features of the primary lesion that favour malignancy include irregular margins; intermediate-to-high SI on T2-WI (i.e., higher than skeletal muscle); hyperintensity on T1-WI indicating haemorrhage; central necrosis; high signal on high b-value DWI (i.e., equal to or higher than that of the endometrium or LNs in the non-necrotic portion); and corresponding low ADC values [26, 29]. These features are illustrated in Fig. 6. In recent years, emphasis has been placed on quantifying ADC values of solid enhancing tissue, with one study proposing a cut-off of < 0.9 × 10⁻³ mm²/s as suspicious for malignancy [30].

More recently, clinical features such as menopausal status have been incorporated alongside imaging features to further improve diagnostic accuracy, although additional studies are needed [27].

## Summary statement

MRI is the gold standard for assessing uterine malignancies, with indications ranging from staging and treatment planning to detection of local recurrence. FDG-PET/CT is typically used for the assessment of LN and distant metastases, particularly in CC, while CT is generally reserved for distant staging or follow-up after treatment. Adherence to recommended imaging protocols is essential for accurate interpretation and evaluation of key tumour-related parameters that may influence subsequent management. Depending on the tumour type, specific imaging features should be systematically assessed and reported to ensure clinicians receive the necessary information for appropriate therapeutic decision-making.

## Patient summary

MRI is the main imaging test used to evaluate cancers of the uterus. It helps radiologists determine how far the cancer has spread, plan treatment, and check for recurrence. FDG-PET/CT, which identifies areas of increased metabolic activity, is often added to assess LNs and distant metastases, while CT is mainly used to detect distant spread or during follow-up. Adherence to specific imaging guidelines ensures radiologists provide accurate, clinically relevant information to guide patient care.
